# Unlabeled topical anesthetic-induced severe methemoglobinemia in a pediatric burn patient

**DOI:** 10.1093/jscr/rjaf334

**Published:** 2025-06-16

**Authors:** Christopher Mahir, Christopher Bean, Gennadiy Fuzaylov

**Affiliations:** UMass Chan Medical School, Department of Anesthesia and Perioperative Medicine, 55 N Lake Ave, Worcester, MA 01655, United States; Massachusetts General Hospital, Department of Anesthesia, Critical Care and Pain Medicine, 55 Fruit Street, Boston, MA 02114, United States; Massachusetts General Hospital, Department of Anesthesia, Critical Care and Pain Medicine, 55 Fruit Street, Boston, MA 02114, United States

**Keywords:** methemoglobinemia, medication labeling, telemedicine, topical anesthetic, burn injury

## Abstract

This case report presents a patient with acquired methemoglobinemia due to unlabeled local anesthetic use. Some local anesthetics disrupt oxidative phosphorylation in erythrocytes, forming methemoglobin, which cannot transport oxygen. A 10-year-old boy with 60% total body surface area flame burns experienced sudden deterioration on Day 6 in the pediatric intensive care unit, presenting with cyanosis, tachycardia, hypotension, and SpO2 of 76% on room air, improving to 90% on 100% FiO2. A telemedicine consultation was requested, and evaluation revealed an elevated methemoglobin level of 22%. The source was an inadequately labeled ointment containing prilocaine used for burn wound care. This case underscores the importance of vigilant monitoring when using local anesthetics on pediatric burn patients. Additionally, global telemedicine played a crucial role in the timely diagnosis and management of this rare but life-threatening condition.

## Introduction

Medication safety remains a pressing global health issue, particularly in resource-limited settings where regulatory inconsistencies and inadequate labeling contribute to preventable adverse events. In many low- and middle-income countries, unstandardized medication packaging and insufficient pharmacist oversight increase the risk of medication errors, leading to serious patient harm. Telemedicine has emerged as a critical tool in bridging healthcare disparities by providing access to specialized expertise and improving patient outcomes in under-resourced areas.

This case report illustrates these challenges through the experience of a 10-year-old boy with extensive flame burns who developed life-threatening methemoglobinemia due to an unlabeled topical anesthetic. Despite aggressive supportive care, his condition worsened until a telemedicine consultation with a US physician led to the identification of the condition and appropriate treatment. The rapid response and successful recovery emphasize the necessity of standardized medication labeling, increased awareness of local anesthetic toxicity, and the role of telemedicine in enhancing patient safety and equitable healthcare access worldwide.

## Case report

A 10-year-old boy suffered flame burns with a total body surface area of up to 60% and was treated in the Regional children’s hospital intensive care unit in Ukraine. The child’s condition acutely deteriorated with central cyanosis, severe tachycardia, arterial hypotension, and a decrease in SpO2 to 76% despite maximum therapy including intubation and ventilation with 100% FiO2. A consult was made to a US physician via telemedicine given the relative lack of expertise in pediatric burn patients in the region. During systematic exam and laboratory evaluations of the child’s hypoxemia, we discovered the methemoglobin level was elevated to 22%. Diagnosis of methemoglobinemia was made and treatment administered. To establish the cause of methemoglobinemia, we conducted a complete analysis of medications the patient had received. The source of intoxication was discovered to be an unlabeled ointment containing prilocaine that is used routinely on burn surfaces as a local wound treatment. A complete recovery was made following cessation of the application of the ointment and appropriate therapy with methylene blue 2 mg/kg over the span of 30 minutes. The patient gradually improved and stabilized within two hours. After a 45-day hospital stay, the patient was discharged home.

## Discussion

In the previously mentioned case, a child nearly suffered a fatal reaction due to an unlabeled topical medication containing a local anesthetic, leading to methemoglobinemia. This preventable adverse reaction underscores the critical need for stricter guidelines and monitoring to ensure proper medication labeling, particularly in settings where regulatory oversight may be insufficient.

Although this patient had a favorable outcome due to prompt recognition and treatment, similar cases may not be as fortunate, especially in lower-middle-income countries where access to treatment is limited. The lack of standardized medication safety guidelines across different nations highlights disparities in healthcare resources and regulatory enforcement. In some regions, inadequate monitoring and inconsistent regulations increase the risk of medication errors, contributing to patient harm and avoidable healthcare costs.

Many developing countries lack sufficient safeguards for medicine naming, labeling, and packaging, often failing to account for human error in drug selection and design. Studies indicate deficiencies in pharmacy services, often lacking trained personnel, which results in incorrect dosages or misidentified medications [1]. Furthermore, patients frequently receive medications without understanding their contents or potential risks, as seen in our case. This highlights the importance of promoting patient awareness and engagement in their healthcare decisions.

Proper medication labeling should include essential information such as the generic and brand names, dosing, strength, administration route, and warnings. It is crucial that this information is presented in a standardized, clear, and accessible manner on packaging. While individual countries may have specific labeling requirements, efforts should be made to establish greater consistency both within and between nations to improve safety and reduce medication-related errors [2]. Addressing these inconsistencies is essential, as medication errors contribute significantly to global healthcare burdens. The WHO estimates that unsafe medication practices result in an annual cost of $42 billion worldwide [3].

Efforts are underway in certain countries to improve medication safety. For example, the Canadian Patient Safety Institute leads the Canadian arm of the WHO’s “Medication Without Harm” campaign, implementing strategies such as medication reviews, the “5 Questions to Ask About Your Medications” program, and opioid stewardship initiatives to enhance pain management and reduce opioid misuse [4]. In Australia, advocacy from pharmacy organizations has successfully urged the government to prioritize medication safety as a national health concern [5]. However, these advancements remain limited in many developing nations, underscoring the need for increased funding, research, and global collaboration to mitigate medication errors and their harmful consequences.

This case also highlights the increasing importance and utility of telemedicine in expanding access to care, particularly in underserved and resource-limited populations. Telemedicine enables timely consultation with specialists, which is especially critical in settings where healthcare providers or subject matter experts are scarce. This model of care benefits not only low-middle-income countries but also rural and remote areas in high-income nations. A literature review conducted during the COVID-19 pandemic found that patients had a 90% satisfaction rate with telemedicine. Reported benefits included improved access to healthcare, more efficient mobilization of medical resources, and increased service utilization. However, challenges remain, including technological barriers, security concerns, low digital literacy, and financial implications for both patients and providers [6].

In conclusion, this case underscores the necessity of careful monitoring of local anesthetics in pediatric burn patients, the importance of recognizing and preventing methemoglobinemia, and the crucial role of telemedicine in improving equitable access to healthcare. Strengthening global medication safety standards and investing in telemedicine infrastructure are essential steps toward reducing preventable patient harm and improving healthcare outcomes worldwide.
